# The Dynamic Reactance Interaction – How Vested Interests Affect People’s Experience, Behavior, and Cognition in Social Interactions

**DOI:** 10.3389/fpsyg.2015.01752

**Published:** 2015-11-27

**Authors:** Christina Steindl, Eva Jonas

**Affiliations:** Department of Psychology, Social Psychology, University of SalzburgSalzburg, Austria

**Keywords:** social interaction, vested interest, mistrust, experience of reactance, aggressive behavioral intentions, biased cognitions, information search

## Abstract

In social interactions, individuals may sometimes pursue their own interests at the expense of their interaction partner. Such self-interested behaviors impose a threat to the interaction partner’s freedom to act. The current article investigates this threat in the context of interdependence and reactance theory. We explore how vested interests influence reactance process stages of an advisor–client interaction. We aim to explore the interactional process that evolves. In two studies, participants took the perspective of a doctor (advisor) or a patient (client). In both studies we incorporated a vested interest. In Study 1 (*N* = 82) we found that in response to a vested interest of their interaction partner, patients indicated a stronger experience of reactance, more aggressive behavioral intentions, and more biased cognitions than doctors. A serial multiple mediation revealed that a vested interest engendered mistrust toward the interaction partner and this mistrust led to an emerging reactance process. Study 2 (*N* = 207) further demonstrated that doctors expressed their reactance in a subtle way: they revealed a classic confirmation bias when searching for additional information on their preliminary decision preference, indicating stronger defense motivation. We discuss how these findings can help us to understand how social interactions develop dynamically.

## Introduction

Buying a coffee at the bakery, chatting with a colleague at the office, meeting a friend, talking to the neighbors… Every single day we interact with people. These interactions require at least two persons who act, react, and in doing so, influence each other. Most of our daily interactions are easy-going. Imagine for example that Mr. Smith is ill and consults Dr. Boston for advice about possible treatment options. The doctor informs Mr. Smith about the advantages and disadvantages of possible medications and listens carefully while he describes his situation and states his needs and fears. Mr. Smith gets a prescription for his preferred medication, thanks the doctor for her advice and feels understood and appreciated. This example demonstrates how positive actions lead to positive responses and further positive actions. Consequently a favorable and trustful relationship-loop can develop.

However, interactions do not always run that smoothly. For various reason they sometimes develop negatively and the positive relationship-loop changes into a conflict-loop. Getting back to our doctor–patient example, imagine that Dr. Boston financially benefits from selling a specific kind of medication, e.g., a depot injection. She overrides the patient’s wishes and needs, talks him into believing that the injection is best for him and reacts with harsh rejection to any other decision. As the doctor is only interested in his own financial benefit, the patient might fear that he may get a wrong medication and may therefore experience a realistic threat, such as an actual danger to his health (see Stephan and Stephan, 2000). However, the doctor’s vested interest can pose an additional threat because the patient may experience a threat to his freedom to decide for himself. The experience of a freedom threat can also happen without any realistic threat present. Imagine for example that not the doctor but the patient would financially benefit from being sold the depot injection because he collaborated with the company which produced the depot. Although the doctor would neither be threatened economically, nor in his health or safety, he could experience the patient’s vested interest as a threat to his freedom. This was demonstrated in an experiment by Wicklund et al. (1970). Participants were asked to rate six pairs of sunglasses and to choose one pair to model in front of a television camera. The chosen pair could then be purchased for half price. Before participants tried the glasses on, the salesperson either said “If you want to buy a pair, I’ll be glad to handle it, since I get a 50% cut off all orders” (vested interest condition) or “I really don’t care whether you buy a pair or not since I don’t get anything from it, but I will be glad to handle the order if you do decide to buy them” (control condition). As participants tried the glasses on, the salesperson pressured them toward a positive evaluation by saying “Those are made for you,” “Those are great.” Findings indicated that the sunglasses were rated lower in the vested interest compared to the control condition. The authors argued that the self-interested behavior of the salesperson created a pressure to buy and thus, as a consequence a desire to maintain the freedom not to purchase any pair arose.

The consequences of social influence attempts like those have been well-explored in research on reactance theory. The theory demonstrates that threats to our freedom to decide for ourselves can lead to a state of motivational arousal with the aim to restore the freedom. This state is known as psychological reactance and has been found to result in different reactance effects. Thus, people show for example aggressive behaviors or biased cognitions such as a devaluation of the imposed object and the threatening person (for an overview on reactance theory, see Brehm, 1966; Brehm and Brehm, 1981; Miron and Brehm, 2006; Steindl et al., 2015).

The aim of the current paper is to improve our understanding of how threats in social interactions lead to conflict loops, i.e., why social influence attempts resulting from vested interests are perceived as threats to freedom and how they lead to the experience of reactance resulting in cognitive and behavioral consequences. We propose a “Loop2Loop model of social interactions” (Jonas, 2015; Jonas and Bierhoff, in press; Jonas and Steindl, 2015), which builds on interdependence theory (Thibaut and Kelley, 1959; Kelley and Thibaut, 1978; Kelley et al., 2003; for an overview see Van Lange and Rusbult, 2011; Van Lange, 2012) and subdivides the interaction process into its single stages. In the present studies we investigate how vested interests in social interactions affect people’s perception of the interaction partner and their subsequent reactions with regard to: (a) their experience of threat, (b) their behavioral intentions, and (c) their cognitions. By analyzing the single stages of the social interaction, which are characteristic for reactance processes, we aim to explain how vested interests shape people’s sequential series of reactions to the threat. In the following paper we refer to this as the “dynamic interaction” between two individuals. This means that in an interaction, person A’s behavior affects person B’s reactions, those again affect person A’s reactions, and so forth.

### Interdependence Theory

Social influence attempts, such as health campaigns or clinical advice, are sometimes perceived as threats to our freedom to decide on our own (e.g., Grandpre et al., 2003; Dillard and Shen, 2005; Silvia, 2005). Those threats often happen in social interactions where one or more persons limit a specific freedom of another person. To understand why and how such negative interactions develop, interdependence theory (Thibaut and Kelley, 1959; Kelley and Thibaut, 1978; Kelley et al., 2003; for an overview see Van Lange and Rusbult, 2011; Van Lange, 2012) may help. Interdependence theory specifies the characteristics of a social exchange situation in which two or more persons are interdependently related to each other. It mainly focuses on the costs and benefits (outcomes) of social interactions.

Interdependence means that the outcomes of the interaction are influenced not only by one’s own behaviors (actor control) but also by the behaviors of the interaction partner (partner control) and by the cooperative behavior of the two partners (joint control). In terms of the theory, we can describe an interaction as a situation in which two people depend on each other with regard to the fulfillment of their needs, thoughts, motives, and behaviors. The more dependent person (high partner control) is likely to sacrifice or accommodate. The less dependent person (low partner control) holds greater power over the other person and threats and coercions are possible (see Van Lange and Rusbult, 2011). For example, Dr. Boston is dependent on the patient’s final choice because she would receive a financial benefit from selling the specific brand of injection to her patient. However, she is less dependent on the patient than vice versa. This could lead the doctor to force the patient to choose the injection. The patient, on the other hand, probably relies even more on the doctor’s behavior (high partner control) than vice versa because he does not have the necessary medical expertise to know which medication is best for him to relieve his symptoms. If the doctor would try “to force” the patient to choose a specific brand of injection, the patient may experience an even stronger feeling of having been threatened, especially if he feels he greatly depends on the doctor’s behavior. In this case, the patient may experience an even stronger threat in response to the doctor’s attempt to influence him compared to a situation in which the patient was less dependent on the doctor’s advice. This asymmetric level of dependence, which means that one interaction partner relies more on the other and thus, is subject to a higher partner control, not only affects people’s satisfaction with the interaction (e.g., Kelley et al., 2003) but further affects their emotional and motivational experience, their cognitions, and behaviors. If the doctor would persuade the patient to take the depot injection, how would the highly dependent patient feel in this situation? And how would those feelings further affect his reactions?

### A Dynamic Model of Social Interaction: The Loop2Loop Model

**FIGURE 1:**
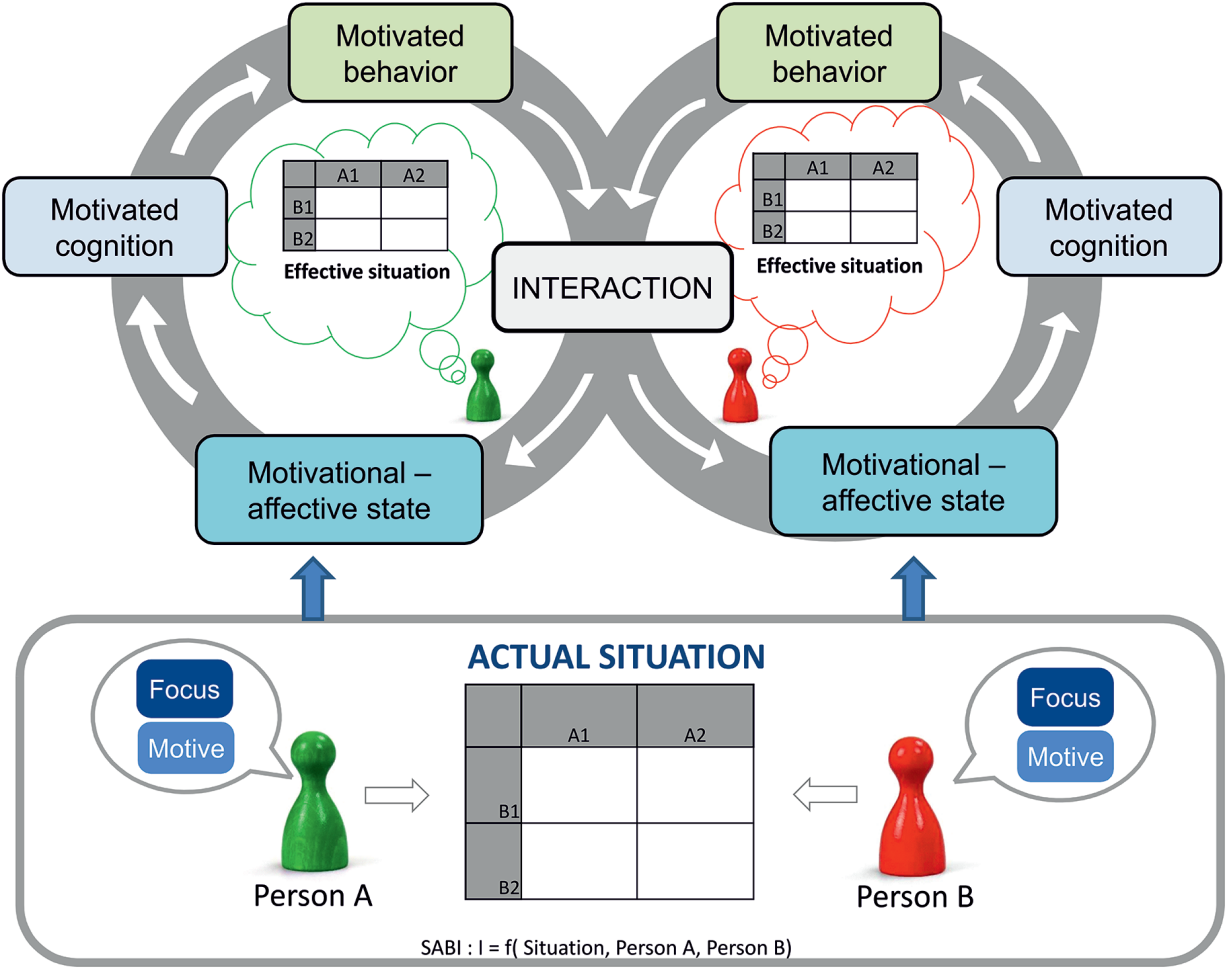
**Loop2Loop model of social interactions (Jonas and Bierhoff, in press)**.

Our “Loop2Loop model of social interaction” (Jonas, 2015; Jonas and Bierhoff, in press; Jonas and Steindl, 2015) describes a dynamic interaction process between two or more persons and how their behaviors mutually affect each other (**Figure 1**). It builds on the SABI model of interdependence theory (Kelley et al., 2003) in which the interaction (I) is a function of two interacting persons’ (A and B) needs, thoughts, motives, and behaviors, and the characteristics of the situation (S): I = *f* (A, B, S). The Loop2Loop Model sets a strong focus on people’s motives influencing people’s motivation for the actual interaction. They give rise to the emerging motivational-affective state, the motivated cognitions, and motivated behaviors. These effects create a dynamic development of social interactions which means that the behavior of one person affects the experience, behaviors, and cognitions of another person which in turn affect the first person and so forth. Therefore, the Loop2Loop model extends the SABI model by explaining how interactions dynamically develop over time. For example, trusting behaviors such as honesty or reliability create a trustworthy atmosphere further arousing a feeling of one’s motives being in good hands. The relationship-loop can develop in a favorable way. On the contrary, untrustworthy behaviors such as cheating or lying threaten one’s motives, arouse a feeling of being threatened and lead to negative cognitions about the interaction partner which further creates a conflict-loop. Jodlbauer and Jonas (2011), for example, found that clients who recognized self-interested cues of advisors perceived the advisors as less trustworthy which in turn decreased their willingness to accept the recommendation. If we would take any further, this could in turn threaten the advisor’s motivation to sell a product and thus, could trigger anger and even aggressive behavior in the advisor. When integrating this example into the Loop2Loop model, we have the advisor’s self-interested behavior in one loop and the client’s resistant behavior (refusal of the recommendation) in the other loop. What about the motivation and cognition in-between? Why do situations involving self-interested behaviors often elicit resistance?

### Vested Interest, Reactance, and Mistrust

Think back to the doctor–patient example in which Dr. Boston tries to persuade her patient to take the depot injection. How would the patient react? Research in the context of persuasion has shown that persuasive attempts, for example, trying to convince a person of a specific opinion or product often fail to produce their desired effects. Rather, they often lead to the exact opposite attitude or behavior. Several studies found psychological reactance to be a possible explanation for these failures (e.g., Worchel and Brehm, 1970; Heller et al., 1973; Dillard and Shen, 2005; Kim et al., 2013; Rains, 2013): Perceived intent to persuade is often perceived as a threat to one’s freedom to decide for oneself leading to a motivation to restore one’s freedom called psychological reactance (Brehm, 1966).

Reactance consists of an affective, as well as a cognitive and a behavioral component. Miron and Brehm (2006) defined the affective component of reactance as one’s subjective experience (feeling) that accompanies the urge to restore freedom. This subjective experience was specified as the experience of uncomfortable, hostile, and aggressive feelings (Brehm, 1966; Brehm and Brehm, 1981), but also as anger affect (Dillard and Shen, 2005). Other research combined the extent to which one perceived a situation as a freedom threat with one’s emotional experience (e.g., frustrated, annoyed, offended) to assess the subjective experience of reactance (Traut-Mattausch et al., 2008, 2011; Jonas et al., 2009; Sittenthaler et al., 2015; Niesta-Kayser et al., submitted). Dillard and Shen (2005) demonstrated that reactance not only consists of affect but also of cognitions. In their persuasion research, they focused on negative cognitions such as counterarguments or derogation of the source of threat. Another cognitive indicator of reactance used in various reactance studies, is the change in attractiveness which means that people upgrade a restricted but downgrade an imposed message or product (e.g., Brehm, 1966; Bushman and Stack, 1996; Fitzsimons and Lehmann, 2004; Dillard and Shen, 2005; Rains and Turner, 2007; Bijvank et al., 2009; Laurin et al., 2012; Rains, 2013). The behavioral component of reactance is often described as the execution of the restricted behavior. In addition, forcing the threatening agent to remove the threat or behaving in a hostile and aggressive way (Brehm, 1966; Brehm and Brehm, 1981; Miron and Brehm, 2006) represents the behavioral component.

However, these reactance effects only emerge if two conditions are met. First, only if people expect to have a certain freedom of choice, reactance can occur (Brehm, 1966; Brehm and Sensenig, 1966; Clee and Wicklund, 1980). Imagine the patient again, who expects to receive an advice from Dr. Boston but still expects being able to decide what is best for him. Second, only if there is a perceived threat to this freedom of choice, reactance can occur. According to Brehm and Sensenig (1966, p. 703), “When a person is free to choose between two alternatives, any attempt by another person to influence his choice should be perceived as a threat to his freedom.” Thus, if the patient notices that Dr. Boston tries to influence him, the patient perceives a threat to his freedom.

When people try to influence someone, they may have manifold reasons for it. One reason for a social influence attempt results from a vested interest. In general, a vested interest is defined as a hedonically relevant attitude object which has important perceived personal consequences for the attitude holder (Crano and Prislin, 1995). The more important the attitude object, the more likely is the attitude expressed in one’s behavior. In many cases, this vested interest is of financial nature. A study by Jones et al. (2014) explored how the explicit mentioning of a financial interest of a salesperson influenced the clients’ satisfaction with their purchase. They manipulated vested interest by salespersons urging customers to evaluate their purchase most positive in order to minimize the risk of decreasing the salesperson’s compensation. If this vested interest was present, participants indicated lower satisfaction with their purchase than if no vested interest was present. Similarly, Wicklund et al. (1970) demonstrated that sunglasses sold by a salesperson with a financial interest were liked less than sunglasses sold by a salesperson with no financial interest. Thus, people’s perception of a vested interest of a salesperson resulted in downgrading the purchase. Such a change in the attractiveness of an object, opinion, or message is an important cognitive indicator of reactance (e.g., Brehm, 1966; Brehm and Brehm, 1981; Laurin et al., 2012). In the studies described above (Wicklund et al., 1970; Jones et al., 2014), people showed reactance after they had perceived a vested interest of their interaction partner.

As research on attitude change suggests, people with vested interests are perceived as less trustworthy (for an overview see Eagly and Chaiken, 1993). In two studies, Jodlbauer and Jonas (2011) found that clients, who perceived self-interested intentions of their advisors, trusted them less which in turn decreased their willingness to accept the advice. Thus, we expect mistrust to explain why vested interests arouse a feeling of being threatened and why this feeling results in reactance effects.

### The Present Research

In the present studies we explore how self-interested behavior can affect the dynamics of social interactions. Therefore, we split up the reactance process into its single stages (experience of reactance, aggressive behavioral intentions, and biased cognitions in the form of negative attitudes toward the threatener and a change in attractiveness of the imposed and non-imposed options). In two studies we explored these stages in an advisor–client context by presenting a fictitious doctor–patient paradigm to participants in which they imagined themselves being in the position of either a patient who sought medical advice from a doctor or being in the position of a doctor treating a patient. In the *neutral paradigm* the interaction partner (either the doctor or the patient) was very open to any kind of medication. In the *vested-interest paradigm*, the interaction partner (either the doctor or the patient) had a financial interest and forced the other person to take/recommend the depot medication. We predicted that after reading the vested-interest paradigm, people would show more mistrust, reactance-related experience, behavior, and cognition than after reading a neutral paradigm. In addition, we predicted that people in the role of a client would react with stronger reactance than people in the role of an advisor. In their role as experts advisors are usually less dependent on the clients than the clients are on them. That is clients are subject to a higher partner control and thus, advisors have power over the clients (Thibaut and Kelley, 1959; Kelley and Thibaut, 1978; Kelley et al., 2003). In the example of the doctor–patient interaction, the patient would suffer more than the doctor from a vested interest because the patient’s health depends on the doctor’s advice. Thus, the patient experiences a realistic threat – an actual danger to his health. A vested interest may additionally be experienced as a threat to one’s freedom to decide for oneself. This should result in a higher defensiveness of a patient compared to a doctor. In addition, if advisors do not have any incentive to appear in a positive light, they seem to be more accuracy-motivated and thus, not as biased as clients or personal decision makers (Jonas and Frey, 2003; Jonas et al., 2005). Therefore, a doctor should feel less affected by a patient’s attempt to threaten the doctor’s freedom.

In both studies, treatment of the participants was in accordance with the ethical standards of the American Psychological Association (APA). Participants were informed that there were no right or wrong answers to the questions, that the data would be treated confidentially and anonymously, and that drawing any personal reference from it would not be possible. In order to assure anonymity, participants were not asked for information that allows inferences to the participants (e.g., names). Participants were aware that they could withdraw from the online-study at any time. Participants were also provided with the name and email address of the responsible investigator. At the end of the survey, participants were thanked for their participation and provided with contact details if they wished to address any questions about the purpose of the study. They also received course credits if desired.

## Study 1

In Study 1 we investigated individuals’ mistrust toward their interaction partner, their experience of reactance, their aggressive behavioral intentions, and their biased cognitions in a fictitious doctor–patient interaction. We further examined whether mistrust toward their interaction partner causes the emerging reactance process. Therefore, we developed scenarios in which people imagined being in the position of a protagonist whose interaction partner shows self-interested behavior. We address the following assumptions:

### Mistrust and Reactance – Main Effects

Firstly, we predicted that after a vested interest of the interaction partner, one perceives the partner as highly untrustworthy, i.e., s/he experiences strong *mistrust* toward the interaction partner (see Eagly and Chaiken, 1993; Jodlbauer and Jonas, 2011). Second, we hypothesized that a vested interest is experienced as a threat to one’s freedom (see Wicklund et al., 1970; Jones et al., 2014). This means that, compared to no vested interest, a vested interest leads participants to *experience more reactance*, reveal more *aggressive behavioral intentions*, and show more biased cognitions, i.e., they have more *negative attitudes* toward their interaction partner and rate the issue that has been imposed on them *less attractive*.

### Reactance – Interaction Effects

As people in the role of clients are first, subject to a higher partner control (see Thibaut and Kelley, 1959; Kelley and Thibaut, 1978; Kelley et al., 2003) and second, have been shown to defend their own position more strongly than people in the role of advisors (see Jonas and Frey, 2003), we predicted that in the current study patients who face a vested interest of their doctor *experience more reactance*, reveal more *aggressive behavioral intentions*, and have more *biased cognitions* than doctors who face a vested interest of their patient.

### Dynamic Development – Mediation Effects

Mistrust should instigate a reactance process to evolve. Therefore, we predicted that people’s perception of *mistrust* mediates the relationship between a vested interest and people’s *experience of reactance*, which further shades into *aggressive behavioral intentions* and finally results in people’s long-term *biased cognitions*. Summarized, the single threat stages should cause the development of the dynamically developing reactance process.

## Materials and Methods

### Participants and Design

Eighty-two students (57 women, 24 men, 1 unspecified; *M*_age_ = 23.84 years, *SD* = 7.18) from the University of Salzburg were asked to read one of four paradigms in which they either were asked to empathize with the role of a doctor (advisor) or with the role of a patient (client). They received either a scenario in which their interaction partner showed a vested interest or a neutral scenario. Thus, the experiment was based on a two (interest: vested interest vs. neutral) × two (role: doctor vs. patient) between-subjects design.

### Materials and Procedure

The online questionnaire first gave general information about the study and obtained some demographic information. After reading short information about schizophrenia, participants rated two types of medication (depot injection vs. pills) for the treatment of schizophrenia in their attractiveness [scale from 1 (*not at all attractive*) to 10 (*very attractive*)]. Participants were randomly assigned the role of a doctor or a patient and asked to read one of two paradigms. Afterwards, they answered questions concerning their mistrust toward the interaction partner, their experience of reactance, their aggressive behavioral intentions, and their biased cognitions [scales from 0 (*not at all attractive*) to 10 (*very attractive*)]. At the end of the survey, participants were thanked for their participation and provided with contact details if they wished to address any open questions about the purpose of the study. They also received course credits if desired.

#### Paradigms

The paradigms consisted of a consultation situation. One group of participants empathized with the *role of a doctor* who had to decide between recommending either a depot injection or pills for treating their schizophrenia patient M. Schneider. In the *neutral scenario* patient M. Schneider was described as being very friendly and open to information about both types of medication. In the *vested-interest scenario* patient M. Schneider was only interested in being recommended the depot. He mentioned that he collaborated with a pharmaceutical company which produced the depot and that he would therefore have financial advantages. He forced participants in the role of the doctor to recommend the depot. Thus the consulting seemed more like a justification of M. Schneider’s already taken decision.

The other group of participants empathized with the *role of the schizophrenia patient* M. Schneider who had to decide which type of medication (depot injection vs. pills) he would choose. In the *neutral scenario* the doctor Dr. Müller was described as very friendly and explained the advantages and disadvantages of both types of medication. In the *vested-interest scenario* Dr. Müller was only interested in recommending the depot. He mentioned that he collaborated with the pharmaceutical company producing the depot and that he would therefore have financial advantages.

#### Questionnaire – Reading Check, Mistrust, and Reactance

To check whether people had read the scenarios thoroughly we assessed whether they ascribed to the doctor/patient a personal interest in recommending/receiving the depot or the pills (*reading check – personal interest depot*; “Do you believe the doctor/patient is personally interested in recommending/receiving the depot?”; *reading check personal interest pills:* Do you believe the doctor/patient is personally interested in recommending/receiving the pills?”). After the paradigm we assessed participants’ *mistrust* toward their interaction partner (seven items; α = 0.90; e.g., “Do you think that the doctor/patient has made his or her decision already before the consultation?”). Then, participants indicated their *experience of reactance*^1^ (Sittenthaler et al., 2015; four items; α = 0.96; e.g., “To what extent do you perceive the behavior as a restriction of freedom?,” “How much does the doctor’s/patient’s behavior bother you?”). We also assessed people’s *aggressive behavioral intentions* (three items; α = 0.84; e.g., “How likely are you to describe this doctor as incompetent to other patients?/How likely are you to describe this patient as stubborn to colleagues?”). To assess participants’ biased cognitions, we asked them to indicate their attitude toward the interaction partner (*negative attitudes*; two items, r = 0.46^2^; e.g., “Do you think that this doctor/patient could have prejudices against mentally ill people?”). Moreover, before and after participants read the scenarios, we assessed the *attractiveness* of the depot and pills medication. For analyzing people’s judgment of attractiveness of the imposed and non-imposed medication, we took the difference score between attractiveness of the depot or the pills after minus before reading the scenario with negative scores indicating a lower attractiveness of the medication after the scenario.

## Results

### Reading Check

To assure that participants had read the scenarios thoroughly, we computed a difference score between personal interest in depot and personal interest in pills with positive scores indicating a higher interest in the depot compared to the pills medication. The univariate analysis of variance (ANOVA) with interest (vested interest vs. neutral) and role (doctor vs. patient) as independent variables and the difference score as dependent variable showed a significant main effect for interest, *F*(1,78) = 94.82, *p* < 0.001, η^2^ = 0.55. Participants in the vested-interest condition who had been imposed the depot were indeed more strongly convinced that their interaction partner was personally interested in recommending or receiving the depot over the pills (*M* = 6.63, *SD* = 2.68), while participants in the neutral condition were convinced that their interaction partner was more or less equally interested in both medications (*M* = 1.20, *SD* = 2.46). Thus, we can conclude that people realized the threatening partner’s intentions. Furthermore, the main effect for role was significant, *F*(1,78) = 4.63, *p* = 0.034, η^2^ = 0.06, indicating that participants in the role of patients were in general more strongly convinced that the doctor was personally interested in recommending the depot over the pills (*M* = 4.50, *SD* = 3.90) than vice versa (*M* = 3.30, *SD* = 3.52). The interaction between interest and role was not significant, *F*(1,78) < 1, *p* = 0.444, η^2^ < 0.01.

### Mistrust and Reactance

We tested our predictions that first, threatened participants experience strong *mistrust* toward the interaction partner, second, that in general threatened participants show more reactance (experience of reactance, aggressive behavioral intentions, negative attitudes) than non-threatened participants, and third, that especially participants in the role of threatened patients experience the most reactance (experience of reactance, aggressive behavioral intentions, negative attitudes). We ran a multivariate analysis of variance (MANOVA) with interest (vested interest vs. neutral) and role (doctors vs. patients) as independent variables and mistrust, the three reactance scales, and the two attractiveness ratings as dependent variables.

First, for mistrust, the analyses revealed a significant main effect for interest, *F*(1,78) = 268.95, *p* < 0.001, η^2^ = 0.78. This indicates that participants in the vested-interest group perceived more mistrust toward their interaction partner than participants in the neutral group (*M* = 8.37, *SD* = 0.97 vs. *M* = 4.06, *SD* = 1.56) which supports our hypothesis. Furthermore, the main effect for role was significant, *F*(1,78) = 16.58, *p* < 0.001, η^2^ = 0.18, indicating that in general, participants in the role of patients perceived more mistrust than participants in the role of doctors (*M* = 6.74, *SD* = 2.44 vs. *M* = 5.67, *SD* = 2.51). The interaction between interest and role was not significant, *F*(1,78) < 1, *p* = 0.491, η^2^ < 0.01.

Second, for reactance, the MANOVA revealed significant main effects for the factor interest on the scales *experience of reactance*, *F*(1,78) = 101.06, *p* < 0.001, η^2^ = 0.56, *aggressive behavioral intentions*, *F*(1,78) = 33.61, *p* < 0.001, η^2^ = 0.30, *negative attitudes*, *F*(1,78) = 17.76, *p* < 0.001, η^2^ = 0.19, and people’s judgment of the *attractiveness of the depot*, *F*(1,78) = 4.23, *p* = 0.043, η^2^ = 0.05. However, people’s judgment of the *attractiveness of the pills* was not significant, *F*(1,78) < 1, *p* = 0.520, η^2^ = 0.01. Thus, the vested interest evoked more reactance than the neutral condition (*experience of reactance: M* = 6.82, *SD* = 2.67 vs. *M* = 2.36, *SD* = 2.23; *aggressive behavioral intentions*: *M* = 4.20, *SD* = 2.17 vs. *M* = 1.80, *SD* = 1.89; *negative attitudes: M* = 5.98, *SD* = 1.73 vs. *M* = 4.37, *SD* = 1.80; *attractiveness of depot after minus before*: *M* = -0.27, *SD* = 2.68 vs. *M* = 0.88, *SD* = 2.33; *attractiveness of pills after minus before*: *M* = -0.27, *SD* = 2.87 vs. *M* = -0.61, *SD* = 2.17). The results support our hypotheses.

Furthermore, the analyses revealed significant main effects for role on most of the scales, indicating that in general, participants in the role of patients show more *experience of reactance* (*M* = 5.94, *SD* = 3.38 vs. *M* = 3.17, *SD* = 2.61), *F*(1,78) = 39.46, *p* < 0.001, η^2^ = 0.34, more *aggressive behavioral intentions* (*M* = 3.81, *SD* = 2.59 vs. *M* = 2.15, *SD* = 1.75), *F*(1,78) = 16.05, *p* < 0.001, η^2^ = 0.17, and more *negative attitudes* (*M* = 5.60, *SD* = 1.92 vs. *M* = 4.73, *SD* = 1.88), *F*(1,78) = 5.07, *p* = 0.027, η^2^ = 0.06, than participants in the role of doctors. We did not find a significant main effect for role on people’s judgment of the *attractiveness* of both medications, *F*s(1,78) ≤ 1.86, *p*s ≥ 0.176, η^2^s ≤ 0.023.

**FIGURE 2:**
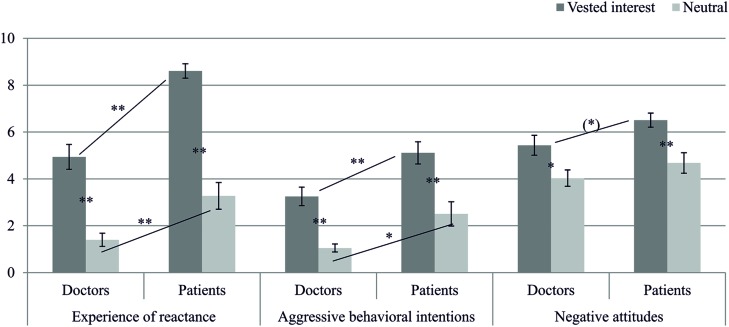
**Means and confidence intervals for participants’ experience of reactance, their aggressive behavioral intentions, and their negative attitudes in Study 1**. Simple-effects are marked with ^∗∗^*p* < 0.01, ^∗^*p* < 0.05, or ^(∗)^*p* < 0.10.

The MANOVA also revealed a significant interaction between interest and role for people’s *experience of reactance*, *F*(1,78) = 4.14, *p* = 0.045, η^2^ = 0.05. Thus, patients in the vested-interest condition experienced the most reactance toward their interaction partner (for detailed results, see **Table 1** and **Figure 2**). However, neither the interactions for people’s *aggressive behavioral intentions* and *people’s negative attitudes*, *F*s(1,78) < 1, *p*s ≥ 0.580, η^2^s < 0.01, nor for their judgment of the *attractiveness* of both medications, *F*s(1,78) ≤ 1.77, *p*s ≥ 0.187, η^2^s ≤ 0.022, were significant.

**Table 1:** Means, standard deviations, and intercorrelations for participants’ mistrust, experience of reactance, aggressive behavioral intentions, and biased cognitions in Study 1.

			*M*	*SD*	1	2	3	4	5	6
(1) Mistrust	All (*N* = 82)		6.22	2.52	–					
	Vested interest	Doctors	7.91	1.07	–					
		Patients	8.80	0.63	–					
	Neutral	Doctors	3.42	1.10	–					
		Patients	4.67	1.70	–					
(2) Experience of reactance	All (*N* = 82)		4.59	3.32	0.81^∗∗^	–				
	Vested interest	Doctors	4.94	2.40	0.19	–				
		Patients	8.61	1.38	0.49^∗^	–				
	Neutral	Doctors	1.40	1.28	0.68^∗∗^	–				
		Patients	3.27	2.57	0.68^∗∗^	–				
(3) Aggressive behavioral intentions	All (*N* = 82)		3.00	2.36	0.64^∗∗^	0.77^∗∗^	–			
	Vested interest	Doctors	3.25	1.78	0.03	0.55^∗^	–			
		Patients	5.11	2.15	0.43	0.22	–			
	Neutral	Doctors	1.05	0.76	0.36	0.72^∗∗^	–			
		Patients	2.51	2.35	0.53^∗^	0.83^∗∗^	–			
(4) Negative attitude	All (*N* = 82)		5.17	1.94	0.52^∗∗^	0.57^∗∗^	0.65^∗∗^	–		
	Vested interest	Doctors	5.43	1.93	0.29	0.15	0.40	–		
		Patients	6.51	1.37	0.53^∗^	0.30	0.48^∗∗^	–		
	Neutral	Doctors	4.03	1.57	-0.11	0.07	0.22	–		
		Patients	4.68	1.98	0.42	0.67^∗∗^	0.74^∗∗^	–		
(5) Change in attractiveness^+^ – depot	All (*N* = 82)		0.31	2.56	-0.19	-0.14	-0.10	-0.12	–	
	Vested interest	Doctors	-0.36	2.32	-0.32	0.33	0.26	-0.26	–	
		Patients	-0.19	3.04	0.25	0.09	0.13	0.15	–	
	Neutral	Doctors	1.10	2.13	-0.01	-0.11	-0.06	0.06	–	
		Patients	0.67	2.54	0.16	-0.22	-0.19	-0.04	–	
(6) Change in attractiveness^+^ – pills	All (*N* = 82)		-0.44	2.53	-0.02	-0.08	0.08	0.19	-0.46^∗∗^	–
	Vested interest	Doctors	0.50	1.99	0.04	-0.21	0.12	0.44	-0.69^∗∗^	–
		Patients	-1.00	3.41	0.12	0.06	0.11	0.26	-0.62^∗∗^	–
	Neutral	Doctors	-0.60	1.47	-0.46^∗^	-0.28	-0.18	0.12	-0.08	–
		Patients	-0.62	2.71	-0.22	-0.03	0.22	0.17	-0.26	–

*Ratings were made on an 11-point scale. ^+^The change in attractiveness values are the difference scores between the ratings made after minus before reading the scenario with negative scores indicating a lower attractiveness of the medication after the scenario. ^∗^*p* < 0.05, two-tailed; ^∗∗^*p* < 0.01, two-tailed*.

### Dynamic Process – Mediation Effects

**FIGURE 3:**
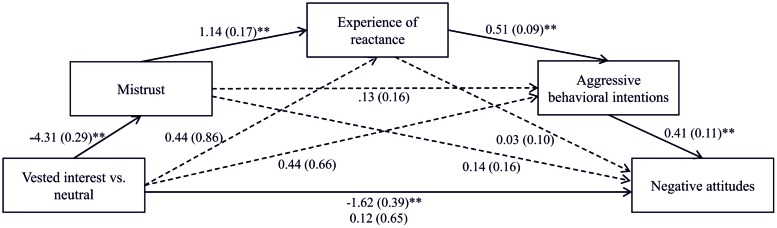
**The effect of freedom threat on negative attitudes via the perception of mistrust, experience of reactance, and aggressive behavioral intentions**. ^∗∗^*p* < 0.01.

For testing the assumption that people’s perception of mistrust causes a dynamic development of a reactance process, we performed a serial multiple mediation analysis using the software Process 2.11 (Hayes, 2013, model 6). The criterion for detecting a serial multiple mediation was a significant indirect effect which was computed using a 99% bias corrected bootstrap confidence interval (99% BCCI) and 10,000 bootstrap samples (Preacher and Hayes, 2008). Regression analyses revealed that the vested interest had a significant total effect on negative attitudes, *b* = -1.62, *SE* = 0.39, *t*(80) = -4.14, *p* < 0.001. The effect decreased to non-significance when the potential mediators mistrust, experience of reactance, and aggressive behavioral intentions had been added to the prediction, *b* = 0.12, *SE* = 0.65, *t*(80) = 0.18, *p* = 0.86. The bootstrapped indirect effect of vested interest on biased attitude via mistrust, experience of reactance, and aggressive behavioral intentions was significant, *b* = -1.04, *SE* = 0.45, BCCI [-2.91, -0.27]. In sum, the perception of mistrust resulting from a vested interest translates into an increased experience of reactance, which in turn leads to more aggressive behavioral intentions and finally results in increased negative attitudes (for the path coefficients see **Figure 3**). This result supports our hypothesis.

## Discussion

In Study 1, our goal was to investigate how individuals in the role of doctors or patients react to vested interests. We examined their mistrust, experience of reactance, aggressive behavioral intentions, and biased cognitions after their interaction partner forced them to choose the depot injection instead of the pills medication. We further aimed to investigate how and why the dynamic in this interaction process develops. First, we found that participants who had been imposed the depot, indicated more mistrust than participants in a control group. Second, a serial multiple mediation revealed that the vested interest caused a chain of reactance reactions because it engendered mistrust toward the interaction partner. This suggests that the perception of mistrust significantly contributes to the person’s experience, behaviors, and cognitions and therefore to a dynamically developing conflict-loop.

Maybe even more interesting, patients who had been imposed the depot partly indicated a stronger experience of reactance, more aggressive behavioral intentions, and more biased cognitions than doctors who had been imposed the depot. Derived from interdependence theory (Thibaut and Kelley, 1959; Kelley and Thibaut, 1978; Kelley et al., 2003), we suggest that this result can be explained by the different partner control of doctors and patients. Patients are usually more dependent on the doctors than vice versa. This would mean that patients experience a stronger threat if they perceive the doctor being only interested in his/her own financial benefit but not in the patients’ health. Moreover, advisors and clients have different roles. People who are in the role of advisors may not be as concerned with their own decisions but may simply make a professional recommendation for the client (Jonas and Frey, 2003). In doing so maybe they try to focus more on their clients’ wishes and needs than on their own. Thus, when threatened by their patients, participants in the role of a doctor might demonstrate less reactance what is important for the further development of a trustful advisor–client relationship. This may be the reasons why participants in the role of advisors seem to behave in a less defensive manner than clients when confronted with a threat.

However, is it really true that participants in the role of advisors are less defensive than participants in the role of clients? Or were our explicit measures just inappropriate to detect their real reactions? What happens if we incorporate a more implicit or indirect measure to detect reactance among participants in the role of an advisor?

## Study 2

In Study 2 we aimed to replicate the results of Study 1 indicating that advisors show less reactance than clients when using explicit measures. In Study 1 we could also show that threatened participants downgraded the depot which indicates a lower interest in the imposed medication. This change in people’s interest may also affect their kind of information search. Vested interests might also lead people to become interested in learning more about or reading more about the advantages than the disadvantages of the restricted option and they might also become more interested in further information about the disadvantages than about the advantages of the imposed option. Thus, Study 2 also included a more indirect measure of reactance, people’s *information search*.

Research has found that human information search is often biased (Frey, 1986; Jonas et al., 2001). If people have made a choice, they prefer information supporting their decision over information opposing their decision, i.e., they reveal a “confirmation bias” (Jonas et al., 2001, p. 557). This phenomenon has been explored in the context of dissonance theory (Festinger, 1957). Research suggests that when people make decisions for themselves, they preferentially search for information supporting their preliminary decision, which serves to reduce post-decisional conflict. With regard to the information search among advisors, research suggests that if advisors are led by accuracy motivation they consider information supporting and contradicting their initial recommendation in a more balanced way (Jonas and Frey, 2003; Jonas et al., 2005). However, the confirmation bias increases for advisors if there is an incentive to justify their position or appear in a positive light to the client (Jonas et al., 2005). If advisors, on the other hand, are motivated to avoid a wrong decision, they reveal another kind of bias: they consider information contradicting their position more carefully and even exhibit a disconfirmation bias (Jonas et al., 2005; Mendel et al., 2009). Investigating the information search of psychiatrists when considering the diagnosis of a patient, Mendel et al. (2009) found that doctors informed themselves more about the risks than about the benefits of available treatment options. This effect can be explained by the doctor’s principle of “primum non nocere” which is the motivation of avoiding harm (Mendel et al., 2009) and indeed, the results showed that the more the doctors investigated conflicting information the higher the probability that they made the correct diagnosis, whereas the probability of sticking to their wrong decision was highest when they revealed a confirmation bias (see also Kray, 2000).

We presented participants with information about the facts of the imposed and non-imposed options and with information about the person who showed a vested interest. Firstly, we predicted that due to their professional role, doctors would search for more information overall than patients (Kray, 2000; Mendel et al., 2009). Secondly, we were interested in whether doctors who are in a more powerful role than clients express their experienced reactance in a more subtle way when they have been facing a vested interest of their interaction partner. Thus, we wanted to know if they show a confirmation bias in information search by preferring supporting information about their initially recommended medication and non-supporting information about the imposed medication.

## Materials and Methods

### Participants and Design

We had 225 participants who participated for course credit in an online study. However, we needed to exclude the data of seven participants because they correctly inferred the manipulation and eleven because they indicated that they had been unable to identify with the doctor or the patient in the scenario. Our final sample consisted of 207 students of the University of Salzburg (143 women, 62 men, 2 unspecified; *M*_age_ = 25.07 years, *SD* = 6.31). The experiment was based on a two (interest: vested interest vs. neutral) × two (role: doctor vs. patient) between-subjects design. Participants were instructed to take the role of a doctor (advisor) or a patient (client) and were asked to read one of four consultation paradigms described in Study 1.

### Materials and Procedure

We again assessed participants’ experience of reactance, their aggressive behavioral intentions, and biased cognitions using the same items as in Study 1. Additionally to assess whether participants searched for more information supporting vs. conflicting with their initial decision preference, at the beginning of the questionnaire we asked them to indicate their spontaneous decision for one of the two medications (depot vs. pills). After the questions regarding their experience of reactance, aggressive behavioral intentions, and biased cognitions we presented them information concerning the treatment options and participants indicated whether they wanted to read it in more detail or not (see below).

#### Questionnaire – Reactance

Participants answered some items regarding their experience of reactance (four items, α = 0.96), their aggressive behavioral intentions (three items, α = 0.88), and their biased cognitions (negative attitudes: two items^3^, r = 0.56; attractiveness of the depot and the pills medication before and after the scenario: each one item). All items ranged from 1 (not at all) to 10 (very much).

#### Information Search

Then we presented short statements concerning the advantages and disadvantages of both medications. Each statement was either a factual information or a person-related information about the advantages or disadvantages of the medication (e.g., *factual contra depot*: “A patient survey showed: the depot injection is often perceived as unpleasant and as evoking anxiety.”; *person-related (doctor) pro depot*: “You hear that patients’ feedback strongly affect Dr. Boston’s work and that so far she has always received positive feedback concerning the depot injection.”). In sum, each participant read sixteen statements (two factual advantages and two factual disadvantages of the depot, two factual advantages and two factual disadvantages of the pills, two person-related (doctor vs. patient) advantages and two person-related disadvantages of the depot, two person-related advantages and two person-related disadvantages of the pills^4^). After reading each statement they indicated whether they wanted to read it in more detail or not. Thus, the information search consisted of the sum of statements they wanted to read more about.

In the end, doctors gave their final recommendation for a medication and patients their final decision for either the depot injection or the pills. They indicated how well they could identify with the role of a doctor/patient, and indicated the objective of the study.

## Results

### Reactance

**FIGURE 4:**
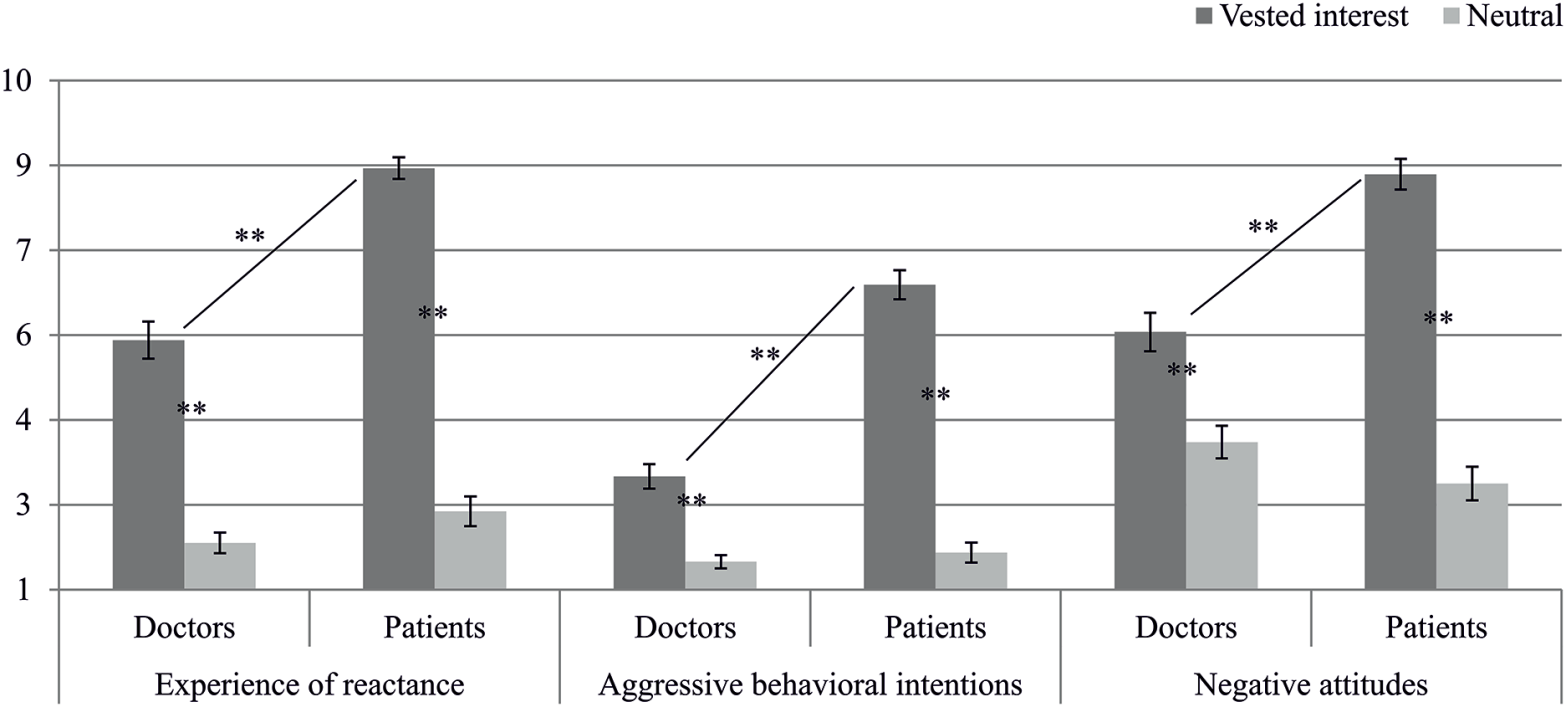
**Means and confidence intervals for participants’ experience of reactance, their aggressive behavioral intentions, and their negative attitudes in Study 2**. Simple-effects are marked with ^∗∗^*p* < 0.01.

Aiming to replicate the results of Study 1, we predicted that individuals, especially patients, who face a vested interest of their interaction partner, reveal more experience of reactance, more aggressive behavioral intentions, and more biased cognitions, than non-threatened individuals. The MANOVA with interest (vested interest vs. neutral) and role (doctors vs. patients) as independent variables revealed significant interactions on all scales except of the attractiveness rating of the pills [experience of reactance: F(1,203) = 24.22, p < 0.001, η^2^ = 0.11; aggressive behavioral intentions: F(1,203) = 61.04, p < 0.001, η^2^ = 0.23; biased cognition – negative attitudes: F(1,203) = 33.20, p < 0.001, η^2^ = 0.14; biased cognition – change in attractiveness depot: F(1,202^5^) = 7.30, p = 0.007, η^2^ = 0.04; biased cognition – change in attractiveness pills: F(1,203) = 1.42, p = 0.234, η^2^ < 0.01]. Thus, we did not only find a significant interaction for experience of reactance like in Study 1, but this time for the other scales as well. Patients who had been imposed the depot indicated the highest reactance. Furthermore, patients also revealed the strongest decrease in the attractiveness of the imposed depot, which confirms our hypotheses (for detailed results, see **Table 2** and **Figure 4**).

**Table 2:** Means, standard deviations, and intercorrelations for participants’ experience of reactance, aggressive behavioral intentions, and biased cognitions in Study 2.

			*M*	*SD*	1	2	3	4	5
(1) Experience of reactance	All (*N* = 207)		4.75	3.23	–				
	Vested interest	Doctors	5.41	2.39	–				
		Patients	8.45	1.38	–				
	Neutral	Doctors	1.83	1.32	–				
		Patients	2.38	1.91	–				
(2) Aggressive behavioral intentions	All (*N* = 207)		3.30	2.50	0.84^∗∗^	–			
	Vested interest	Doctors	3.00	1.59	0.60^∗∗^	–			
		Patients	6.39	1.87	0.54^∗∗^	–			
	Neutral	Doctors	1.49	0.86	0.82^∗∗^	–			
		Patients	1.65	1.29	0.54^∗∗^	–			
(3) Negative attitude	All (*N* = 207)		5.27	3.06	0.75^∗∗^	0.74^∗∗^	–		
	Vested interest	Doctors	5.56	2.48	0.30^∗^	0.27^∗^	–		
		Patients	8.34	1.99	0.50^∗∗^	0.49^∗∗^	–		
	Neutral	Doctors	3.61	2.09	0.46^∗∗^	0.45^∗∗^	–		
		Patients	2.88	2.17	0.64^∗∗^	0.62^∗∗^	–		
(4) Change in attractiveness^+^ – depot	All (*N* = 207)		-0.67	2.26	-0.28^∗∗^	-0.32^∗∗^	-0.21^∗∗^	–	
	Vested interest	Doctors	0.00	2.33	-0.18	-0.17	-0.19	–	
		Patients	-1.95	2.12	-0.24	-0.15	-0.02	–	
	Neutral	Doctors	-0.09	1.74	-0.06	-0.04	-0.13	–	
		Patients	-0.43	2.22	0.14	0.06	0.30^∗^	–	
(5) Change in attractiveness^+^ – pills	All (*N* = 207)		-0.46	2.29	0.04	0.09	-0.07	-0.24^∗∗^	–
	Vested interest	Doctors	-1.00	2.63	0.14	0.04	-0.19	-0.25	–
		Patients	-0.16	2.61	0.11	0.16	-0.11	-0.33^∗^	–
	Neutral	Doctors	-0.39	1.87	-0.13	-0.12	0.01	-0.11	–
		Patients	-0.31	1.75	-0.05	0.12	-0.14	-0.10	–

*Ratings were made on a 10-point scale. ^+^The change in attractiveness values are the difference scores between the ratings made after minus before reading the scenario with negative scores indicating a lower attractiveness of the medication after the scenario. ^∗^*p* < 0.05, two-tailed; ^∗∗^*p* < 0.01, two-tailed*.

### Dynamic Process – Mediation Effects

**FIGURE 5:**
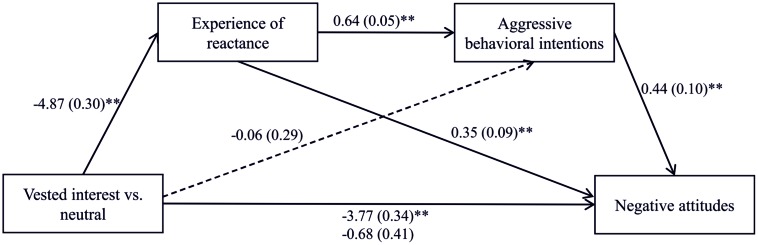
**The effect of freedom threat on negative attitudes via experience of reactance and aggressive behavioral intentions**. ^∗∗^*p* < 0.01.

As the results of the mediation analyses from Study 1 suggest the existence of a dynamic interaction process, we performed a serial multiple mediation analysis (Process 2.11; Hayes, 2013, model 6) also in Study 2. The criterion for detecting the mediation was a significant indirect effect which was again computed using a 99% bias corrected bootstrap confidence interval (99% BCCI) and 10,000 bootstrap samples (Preacher and Hayes, 2008). Regression analyses revealed that the vested interest had a significant total effect on negative attitudes, *b* = -3.77, *SE* = 0.34, *t*(205) = -11.15, *p* < 0.001. The effect decreased to non-significance when the potential mediators experience of reactance and aggressive behavioral intentions had been added to the prediction, *b* = -0.68, *SE* = 0.41, *t*(205) = -1.64, *p* = 0.102. The bootstrapped indirect effect of vested interest on biased attitude via experience of reactance and aggressive behavioral intentions was significant, *b* = -1.36, *SE* = 0.34, BCCI [-2.32, -0.53]. Moreover, the bootstrapped indirect effect of vested interest on biased attitude via experience of reactance alone was also significant, *b* = -1.71, *SE* = 0.48, BCCI [-3.01, -0.53]. In sum, the experience of reactance from a vested interest translates into aggressive behavioral intentions and finally results in increased negative attitudes (for the path coefficients see **Figure 5**). This result again supports our hypothesis of a dynamic development of the reactance process.

### Information Search

First, we tested the prediction that due to their professional role, doctors search for more information than patients. The ANOVA with role (doctors vs. patients) as independent variables and the sum of all information as dependent variable revealed a significant main effect for role on people’s general interest in information, *F*(1,205) = 11.23, *p* = 0.001, η^2^ = 0.06, with doctors searching for more information than patients (*M* = 10.67, *SD* = 4.05 vs. *M* = 8.86, *SD* = 3.72).

Second, we asked whether doctors who are confronted with a vested interest of their patient express their reactance in a subtle way by showing a confirmation bias. According to participant’s spontaneous decision for one of the two medications in the beginning of the study, we ran two separate repeated measures ANOVAs – one analysis for the sample who chose to take the pills and who thus, may have been most affected by the imposition of the depot (*N* = 71, “imposition group”), and one analysis for the sample who chose to take the depot and who thus, may have been feeling confirmed in its decision (*N* = 136, “confirmation group”). To receive a score for the confirmation bias, we calculated a difference value between people’s interest in the number of advantageous and disadvantageous pieces of information chosen.

#### Imposition Group

We inserted interest (vested interest vs. neutral) × role (doctor vs. patient) as the between factors and the differences of the medication (pills vs. depot) × type of information (factual vs. personal) as within factors into a repeated measures ANOVA.

**FIGURE 6:**
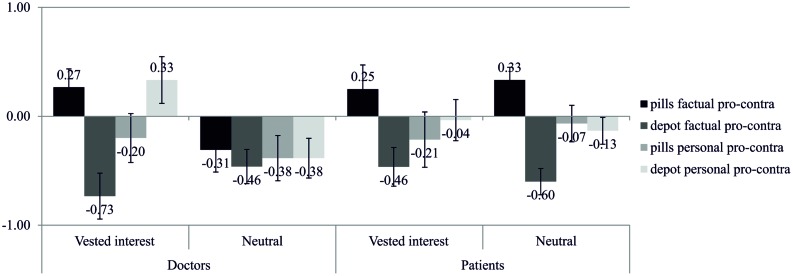
**Information search of participants of the imposition group, i.e., participants who chose to take the pills (*N* = 71) before the scenario**.

The four-way interaction was significant, *F*(1,67) = 4.46, *p* = 0.038, η^2^ = 0.06. Simple effects indicated that doctors in the vested-interest group, i.e., doctors who had been forced to recommend the depot injection (but previously chose to recommend the pills), committed a confirmation bias concerning the factual information: they devalued the imposed depot by showing more interest in the disadvantages than in the advantages of the depot (*M* = -0.73, *SD* = 0.88) but upgraded the non-imposed pills by showing more interest in the advantages than in the disadvantages of the pills (*M* = 0.27, *SD* = 0.70), *p* < 0.001. This upgrading of the pills differed from doctors in the neutral group who devalued the pills by showing a higher interest in disadvantages than in advantages of the pills (*M* = -0.31, *SD* = 0.86), *p* = 0.061 (see **Figure 6**). This means that doctors who had faced a vested interest of their interaction partner more strongly confirmed their decision for recommending the pills than doctors who had not faced a vested interest.

With regard to the patients, both the vested-interest and the neutral group devalued the depot (*M* = -0.46, *SD* = 0.74 and *M* = -0.60, *SD* = 0.51) and upgraded the pills (*M* = 0.25, *SD* = 0.93 and *M* = 0.33, *SD* = 0.49), *p* < 0.001 and *p* = 0.001. This means that, independent of a vested interest of their interaction partner, patients confirmed previously made decisions.

Summarized, a vested interest of the interaction partner only affected doctors but not patients. Doctors reacted to the threat by confirming their previously made decision.

#### Confirmation Group

We inserted interest (vested interest vs. neutral) × role (doctor vs. patient) as the between factors and the differences of the medication (pills vs. depot) × type of information (factual vs. personal) as within factors into a repeated measures ANOVA. The four-way interaction was not significant, *F*(1,132) < 1, *p* = 0.366, η^2^ = 0.01. However, the analysis revealed two significant three-way interactions.

The three-way interaction between role, medication, and type of information, *F*(1,132) = 4.34, *p* = 0.039, η^2^ = 0.03, indicated that concerning the factual information, both doctors and patients were more interested in the advantages of the pills (doctors: *M* = 0.02, *SD* = 0.80; patients: *M* = 0.33, *SD* = 0.94) than in the advantages of the depot (doctors: *M* = -0.52, *SD* = 0.93; patients: *M* = -0.67, *SD* = 0.76), *p*s ≤ 0.001. Furthermore, patients showed a higher interest in the advantages of the pills (*M* = 0.33, *SD* = 0.94) than doctors *(M* = 0.02, *SD* = 0.80), *p* = 0.041. Thus, the advantages of the pills were in general more interesting for people than the advantages of the depot.

The three-way interaction between interest, medication, and type of information, *F*(1,132) = 5.36, *p* = 0.022, η^2^ = 0.04, indicated that concerning the factual information, both the vested-interest and the neutral group were more interested in the advantages of the pills (vested interest: *M* = 0.15, *SD* = 0.92; neutral: *M* = 0.19, *SD* = 0.84) than in the advantages of the depot (vested interest: *M* = -0.44, *SD* = 0.78; neutral: *M* = -0.75, *SD* = 0.91), *p*s < 0.001, and that the neutral group (*M* = -0.75, *SD* = 0.91) was even more interested in the disadvantages of the depot than the vested-interest group (*M* = -0.44, *SD* = 0.78), *p* = 0.037. Thus, the advantages of the pills were in general more interesting for people than the advantages of the depot.

**FIGURE 7:**
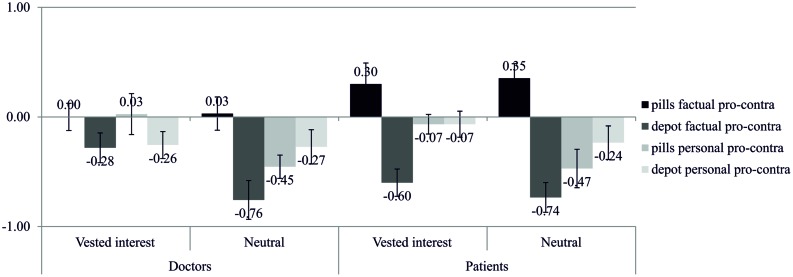
**Information search of participants of the confirmation group, i.e., participants who chose to take the depot (*N* = 136) before the scenario**.

Summarized, simple-effects in the interactions indicate that all groups (doctors, patients, vested-interest, and neutral group) that previously chose the depot showed a higher interest in the factual advantages of the pills than in the factual advantages of the depot (for detailed results, see **Figure 7**). Although they had chosen the depot, they were more interested in the advantages of their non-chosen medication, i.e., the pills.^6^

## Discussion

First, Study 2 showed that patients who had been imposed a medication indicated a stronger experience of reactance, more aggressive behavioral intentions, and more biased cognitions than doctors who had been imposed a medication. These results that partly have also been found in Study 1 suggest that patients are more affected by the vested interest than doctors.

Second, the findings of Study 2 indicate that doctors who have not been imposed a medication, were in general more interested in the disadvantages than the advantages of the medications (see **Figure 6**). Thus, doctors may be careful when it comes to the recommendation of a medication. However, when doctors perceived a vested interest of their patients, they confirmed their previously made decision. They showed a higher interest in the disadvantages of the imposed medication and in the advantages of the non-imposed medication. Thus, doctors behaved like patients who showed this confirmation bias no matter if they perceived a vested interest or not.

Thus, at first sight when only looking at the direct measures of reactance, it seems that doctors who are threatened by a vested interest of their interaction partner are more objective than clients, stay in their professional role, and do not defend themselves. This may be due to the doctors’ professional role in which they are able to distance from the decision problem and are therefore not as impulsive as clients who are concerned with their own decision. Interestingly, Study 2 provides a more differentiated picture compared to Study 1: our data on participants’ information search suggest that doctors in fact show reactance but in a more subtle manner. If the depot had been imposed they demonstrated a classical confirmation bias, i.e., they were more interested in disadvantages of the depot and advantages of the alternative medication. However, this confirmation bias only emerged concerning the factual information but not concerning the personal-related information about the medication and emerged only in doctors and not in patients. Why? Doctors may be more professional if a person is involved. Thus, when it comes to expressing one’s own experience, behaving in a reactant way, or getting informed about the patient, doctors try to remain distanced and professional. Patients, on the contrary, do not have to be professional but can openly express their experience, behavior, and cognition.

## General Discussion

The current research aimed to describe the dynamical development of interactions between two or more persons. With dynamical we mean subdividing the interaction process into its single stages (motivational-affective states, motivated cognitions, and motivated behaviors, see **Figure 1**) and explaining how people’s reactions in an interaction mutually affect each other. The two studies presented here investigated the single stages of an emerging reactance process. After people who imagined being a doctor vs. a patient had been threatened by a vested interest of their interaction partner, we assessed their mistrust toward the interaction partner, their experience of reactance, their aggressive behavioral intentions, and their biased cognitions.

In Study 1 we found that participants who had been imposed a medication indicated more mistrust toward the interaction partner than participants in a control group. A serial multiple mediation revealed that the emerging mistrust triggered a dynamically developing reactance sequence, starting from a high experience of reactance entering aggressive behavioral intentions, and finally ending in people’s biased cognitions. As we found patients being more reactant than doctors we conducted Study 2, in which we aimed to explore the advisor’s weaker reactions to the threat. To do so, in addition to the classic reactance measures of Study 1, we employed an indirect measure of reactance – people’s information search concerning the imposed and the non-imposed medication. The results of Study 2 suggest that doctors show reactance in a subtle way. If the depot had been imposed on them, they were more interested in disadvantages of the depot and advantages of the alternative medication. Patients also showed this confirmation bias but independent of a vested interest. They always confirmed their previously made decisions.

These results suggest that while patients disclose their reactance, doctors show reactance in a more subtle way. The reason for this may be that doctors are less dependent on the patient than vice versa. In terms of interdependence theory (e.g., Kelley et al., 2003), they are subject to less partner control than patients are and thus, have also more power over the patient. The second reason for this may be their professional role, in which they need to remain objective and unbiased. However, especially Study 2 shows us that remaining unbiased after being threatened in one’s freedom is not as easy as it seems. If you take a more thorough look, then even doctors who seem to be very objective at first sight are affected by their patient’s attempt to threaten their freedom.

### Theoretical and Practical Implications

With the current research we tried to investigate how interactions develop dynamically when a threat happens. Although a bunch of studies investigates how threats in social interactions affect motivation, cognition, and behavior, most of them miss the crucial step of linking them and thus showing how they mutually affect each other. However, if we aim to understand how interactions develop, we need to have a look at how one process influences another one. Inspired by interdependence theory (e.g., Kelley et al., 2003), we split the interaction process into its single stages. Going beyond interdependence theory, we further explored how the single stages mutually influence each other. This approach may help to better comprehend why interactions do not always run smoothly but sometimes provoke conflicts.

Following the recommendation of Miron and Brehm (2006, p. 16) to “consider the various implications of the theory for real world phenomena as well as continue revealing and testing its basic theoretical assumptions,” we presented evidence that freedom threats in social interactions may have serious consequences. Therefore, our results are not only important for science but also for our everyday lives. Keeping in mind that threats do not only lead to sudden behavioral reactions but also to long-term biased cognitions, one can better understand own and other’s reactions. This can further contribute to taking action resolving own prejudices against others and other’s prejudices against oneself. Simply knowing how a threat can escalate into a conflict spiral provoking defensive actions and reactions would be the first step for the development of possible interventions.

Although the patient’s behavior is the only part visible for the doctor, the reactant patient additionally has specific cognitions and reasoning processes not visible for the doctor. However, those biased cognitions regarding both, cognitions about the interaction partner and cognitions about oneself and one’s attitudes, are important to consider. Therefore, the patient may derogate the doctor and assume that the doctor has prejudices against all mentally ill people. According to reactance theory, the threatened person (the patient), may also downgrade an imposed medication that he previously preferred and upgrade a not imposed medication that he previously rejected (see also, e.g., Bushman and Stack, 1996; Dillard and Shen, 2005; Bijvank et al., 2009). Our results extend reactance theory by showing that biased cognitions can also be found in threatened people’s information search. We presented evidence that doctors displayed higher interest in information of the non-imposed medication compared to a lower interest in information of the imposed medication.

### Limitations and Future Research

A limitation of our studies is that we used scenarios in which people were imagining being a doctor or a patient. Such imaginations miss out the important real-life setting in which doctors are committed to their profession and patients suffer from a real disease. Although real-life settings would tell us even more, we believe that these studies are first steps into exploring interaction processes developing out of freedom threats. Thus, we found a chain of emerging stages that influenced each other – a threat of one person to another person led to an experience of mistrust, which further aroused an experience of threat and intended behaviors to restore one’ freedom and finally resulted in biased attitudes. Aside from that, it would be difficult and even unethical to manipulate freedom threats in a real doctor–patient interaction. One could only try to observe interactions like these and figure out where threats happen. However, it may be worth to consider carrying out the same studies on a sample of real advisors and real patients and not only on students. Although we found that clients were very reactant if the doctor threatened them in their freedom, this might not apply in all situations. Consider a patient who is really afraid of dying and knows he does not have much medical knowledge. He or she would probably follow the doctor’s orders without experiencing reactance.

Although one would assume that doctors could not be threatened in a realistic way because they do not have a direct benefit from prescribing the depot or not, our results show that doctors are threatened as well but they suffer to a lower degree than patients. The reason for this might be their lower dependence on the interaction partner. This means that the doctor is not as dependent on the patient as vice versa. If the patient, however, recognizes that the doctor is not really interested in the patient’s health but only in her own financial benefit, the patient experiences a very strong threat. Besides his health being threatened, the vested interest of the doctor can also pose a psychological threat. The patient may also feel threatened in his motive of being free to decide for himself. However, as the doctor is not as dependent on the patient, she might not be as affected. Still, the doctor might be threatened in his motive of being free to advice the medication he wants to advice. The result that doctors experience a threat as well is in line with reactance theory stating that social influence attempts targeting a specific individual can pose a freedom threat (Brehm, 1966; Brehm and Sensenig, 1966; Clee and Wicklund, 1980; Brehm and Brehm, 1981). We believe that the doctors’ and patients’ different amount of partner control that might cause the different degrees of threat would be a valuable question for future research.

## Conclusion

Dealing with freedom threats that happen in our everyday interactions, the present research broadens our understanding of how social interactions develop – from experiencing mistrust toward the interaction partner to experiencing a strong threat, to further being motivated to restore one’s scope of action and finally developing biased cognitions. Our results open up possibilities on how social interactions might be viewed in a dynamical sense. Research investigating social interaction may thus pay more attention to this dynamic.

## Conflict of Interest Statement

The authors declare that the research was conducted in the absence of any commercial or financial relationships that could be construed as a potential conflict of interest.

## Footnotes

1 Originally, our reactance measure consisted of 18 items. A study validating this measure (Sittenthaler et al., 2015) reduced the scale to a 10 items measure and confirmed a three-factor structure consisting of *experience of reactance* (four items), *aggressive behavioral intentions* (three items), and *negative attitudes* (three items). In the current article, we only report the validated scales.

2 Due to a reliability of α = 0.58 with three items we excluded the item “Do you think that the doctor/patient shows a similar behavior in other areas?”

3 Because of a bad overall reliability in Study 1, we deleted the item “Do you think that the doctor/patient shows a similar behavior in other areas?”

4 In a pretest we aimed to find clear statements for the dimensions pro depot, contra depot, pro pills, contra pills. Therefore, we asked 30 participants to rate 24 short factual statements, and 20 short person-related (either doctor- or patient-related) statements concerning their direction [scale from -5 (*contra depot/pills*) to 5 (*pro depot/pills*)], persuasiveness, and relevance [scales from 0 (*not at all*) to 10 (*very much*)]. Based on the means we chose eight factual statements and ran 2 (type of medication: depot vs. pills) × 2(information: pro vs. contra) repeated measures ANOVAs with direction, persuasiveness, and relevance separately. For the factual statements, we neither found significant effects for direction, *F*s(1,29) ≤ 2.78, *p*s ≥ 0.107, η^2^ ≤ 0.09, nor for persuasiveness, *F*s(1,29) < 1, *p*s ≥ 0.583, η^2^ ≤ 0.01. For relevance we found a marginal significant effect for information, *F*(1,29) = 3.67, *p* = 0.065, η^2^ = 0.11, indicating that participants ascribing a higher relevance to the pro (*M* = 7.48, *SD* = 0.24) than to the contra information (*M* = 7.10, *SD* = 0.23). For the person-related statements, we neither found significant effects for direction, *F*s(1,13) ≤ 2.74, *p*s ≥ 0.122, η^2^ ≤ 0.17, nor for persuasiveness, *F*s(1,13) ≤ 3.18, *p*s ≥ 0.098, η^2^ ≤ 0.20, or relevance, *F*s(1,13) ≤ 2.01, *p*s ≥ 0.180, η^2^ ≤ 0.13. In sum, the statements did not differ from each other indicating that they were evaluated as clearly speaking for or against the treatment with depot/pills (factual information: *M* = 3.42, *SD* = 0.81; patient-specific information: *M* = 2.60, *SD* = 1.22; doctor-specific information: *M* = 2.05, *SD* = 1.10), were clearly evaluated as persuasive (factual information: *M* = 7.32, *SD* = 1.04; patient-specific information: *M* = 5.79, *SD* = 2.01; doctor-specific information: *M* = 5.22, *SD* = 2.26), and were clearly evaluated as relevant (factual information: *M* = 7.29, *SD* = 1.17; patient-specific information: *M* = 5.87, *SD* = 2.06; doctor-specific information: *M* = 4.43, *SD* = 1.96). Thus, we used these eight statements in the main questionnaire.

5 One missing.

6 As with regard to personal information, people were in general more interested in disadvantages of both medications (pills and depot), we do not report the detailed analyses here.
